# Graphene Oxide: A Perfect Material for Spatial Light Modulation Based on Plasma Channels

**DOI:** 10.3390/ma10040354

**Published:** 2017-03-28

**Authors:** Chao Tan, Xinghua Wu, Qinkai Wang, Pinghua Tang, Xiaohui Shi, Shiping Zhan, Zaifang Xi, Xiquan Fu

**Affiliations:** 1 School of Information and Electrical Engineering, Hunan University of Science and Technology, Xiangtan 411201, China; chaotanhnu@163.com (C.T.); spzhan86@163.com (S.Z.); zfxi@hnust.edu.cn (Z.X.); 2 Faculty of Materials Science and Chemistry, China University of Geosciences, Wuhan 430074, China; 51205879@163.com; 3 Key Laboratory for Microstructural Functional Materials of Jiangxi Province, College of Science, Jiujiang University, Jiujiang 332005, China; 79792239@163.com; 4 School of Physics and Optoelectronics, Xiangtan University, Xiangtan 411105, China; pinghuatang@hnu.edu.cn; 5 Key Laboratory for Micro-/Nano-Optoelectronic Devices of Ministry of Education, College of Computer Science and Electronic Engineering, Hunan University, Changsha 410082, China; shixiaohui@hnu.edu.cn

**Keywords:** graphene oxide, spatial light modulation, plasma channels

## Abstract

The graphene oxide (GO) is successfully prepared from a purified natural graphite through a pressurized oxidation method. We experimentally demonstrate that GO as an optical media can be used for spatial light modulation based on plasma channels induced by femtosecond pulses. The modulated beam exhibits good propagation properties in free space. It is easy to realize the spatial modulation on the probe beam at a high concentration of GO dispersion solutions, high power and smaller pulse width of the pump beam. We also find that the spatial modulation on the probe beam can be conveniently adjusted through the power and pulse width of pump lasers, dispersion solution concentration.

## 1. Introduction

Graphene, a two-dimensional carbon nanomaterial composed of carbon atoms, has a thickness of single atomic layer, the lateral size can reach to microns or even to millimeters. The graphene is characterized by high charge mobility, high thermal conductivity, high intensity, 2.3% visible light absorption and room-temperature quantum Hall effect. In the last few years, graphenes and GO materials have received enormous interest from the scientific community due to their extraordinary mechanical [1,2], electronic [3,4], optical [5,6], and electrochemical properties [7]. The success of graphene applications highly motivates the exploration of other applications. Prashant et al. probe the electron storage and electron shuttling property of GO by anchoring two redox couples [8]. Next, Ambrosi et al. use electrochemically exfoliated graphene and GO for energy storage, they exhibit good capacitive behavior [9,10]. Single layer graphene as high-performance electrocatalysts for water oxidation was recently reported [11]. Besides, its applications in earphones, new barriers, glucose biosensor, fiber lasers, sheet polarizer, and optical isolators have also attracted a lot of attention [12,13,14,15,16].

Optical modulation is a technology for manipulating optical signals in amplitude, phase or polarization, which are widely used in photonic circuits [17,18], optical communication [19,20], neural network imaging [21], and so on. Various methods can be developed for optical modulation. The deployment of graphene on top of a silicon waveguide is an efficient method to make graphene-silicon hybrid devices. Liu et al. first experimentally report a high-performance, waveguide-integrated electroabsorption modulator based on monolayer graphene [22]. Subsequently, dual-graphene, graphene-based slot-waveguide, graphene-based ridge waveguide, graphene-based waveguide integrated dielectric-loaded plasmonic electro-absorption, graphene M–Z polarizer are chosen for optical modulators [23,24,25,26]. Recently, Dalir et al. demonstrated the realization of optical modulator with a 35 GHz modulation speed based on a planar structure with double-layer grapheme [27]. Then, graphene modulators and switches integrated on silicon and silicon nitride waveguide are reported [28].

In this work, we demonstrate a novel method for spatial light modulation based on plasma channels generated in the GO dispersion solution. This is a new application of GO. Firstly, we have successfully synthesized GO from a purified natural graphite through a pressurized oxidation method. The morphology and structure of GO are detailedly characterized by transmission electron microscope, optical microscope, atomic force microscope and Raman spectrometer. Then, we experimentally show the spatial light modulation on the probe beam based on plasma channels generated in the GO dispersion solution. We also analyze some influence factors on the spatial modulation, such as, concentration of GO dispersion solution, power and pulse-width of pump lasers. Finally, we study the propagation characters of modulated beam in the free space.

## 2. Material Preparation and Experimental Setup

### 2.1. Material Preparation and Characterization

#### 2.1.1. Synthesis of GO

In 2004, Geim and Novoselov observed single-layer graphene by the method of microcleaving, in which the single-layer graphene has been peeled off from highly oriented pyrolytic graphite layer after layer using scotch tape. However, due to the defects of low production rate and high randomness, the preparing method of microcleaving is not conducive to practical application and scientific research. In addition to the use of microcleaving method, the preparation of graphenes also includes SiC epitaxial growth, chemical vapor deposition (CVD) and others. The SiC epitaxial method is to remove carbon atoms from monocrystal SiC to reconstruct graphene by high temperature heating in ultrahigh vacuum. Large-area and high-quality graphene of good compatibility in integrated circuit technology can be achieved by SiC epitaxial method. Chemical vapor deposition (CVD) is a process that inflates the high-temperature furnace with atmosphere of high carbon content so that the carbon atoms resolve from the gas and grow into graphene on the metal substrate materials. The controllable growth of graphene can be achieved by controlling the parameters such as substrate type, growth temperature, gas species, flow rate and so on.

Chemical oxidation reduction method shows a unique advantage while the g order of graphene materials are required. In the chemical oxidation reduction method, graphite intercalation compound is obtained at first from graphite as raw material using strong acid treatment in solution, and then graphene is stripped out after oxygen-containing functional group take shape on the surface of graphene while strong oxidant is added in the solution. Large-scale production can be achieved as a result of the cheap raw materials and simple preparation process in this method.

In 1859, Brodie proposed that GO can be produced by using oxidizing agent KClO_3_ to oxidize graphite in fuming nitric acid. This method was improved in 1898 by Staudenmaier, who introduced concentrated sulfuric acid into fuming nitric acid and washed GO by hydrochloric acid and water. The improvement of the experimental process makes the oxidation of GO more fully. So far, the experimental scheme proposed by Hummers in 1958 has been in the most commonly use. In the scheme, graphite is oxidized by potassium permanganate and sodium nitrate in concentrated sulfuric acid, then the residual oxidant is reduced by hydrogen peroxide, and finally the GO is achieved through the treatments of filtration, washing and vacuum dewatering. Tour improved the method of Hummers in the year of 2010. In the solution of concentrated sulfuric acid H_2_SO_4_ and phosphoric acid H_3_PO_4_, potassium permanganate is used as oxidant to oxidize graphite, and then hydrogen peroxide is used to restore the residual oxidant. Compared with the Hummers’ method, the improved method has the following advantages: the reaction process does not release a large amount of heat, the reaction process does not produce toxic or explosive gases, and the reaction products are more fully oxidized. In this paper, GO materials are prepared by the improved method.

Graphite flakes coming from Sigma-Aldrich (cat#332461, ≥150 μm, Shanghai, China) is used as starting material. Firstly, 90 mL concentrated sulfuric acid and 10 mL phosphoric acid are added to the mixture including 1 g of graphite flakes and 5 g of KMnO_4_. Then the mixture is heated to 50 °C on the electric heating magnetic stirrer after mixing evenly, and is kept at this temperature for 12 h under stir. The reaction product is cooled to room temperature and then added to the mixed solution of 100 mL ice water and 1 mL 30% H_2_O_2_. The product is sifted by a metal sieve of 300 μm pore and then is filtered by polyester fiber filter membrane. The filtrate is centrifuged and washed with water, HCl and ethanol, respectively. After filtration and washing, the product is dried in a vacuum oven for 12 h.

#### 2.1.2. Characterization of the GO

Transmission electron microscopy (TEM) images are obtained on a Tecnai G2 F20 S-TWIN microscope (FEI, Hillsboro, OR, USA) with an accelerating voltage of 200 kV. Atomic force microscope (AFM) measurements are carried out in a Bruker Multimode 8 system (BRUKER, Billerica, MA, USA). Raman spectra are obtained by a Witec alpha300R Confocal Raman system (WITec, Ulm, Germany) with the excitation laser of 532 nm in air ambient environment. An optical image is obtained to observe the morphology of the GO by Leica 2700 M optical microscope (Leica, Biberach, Germany).

**Figure 1:**
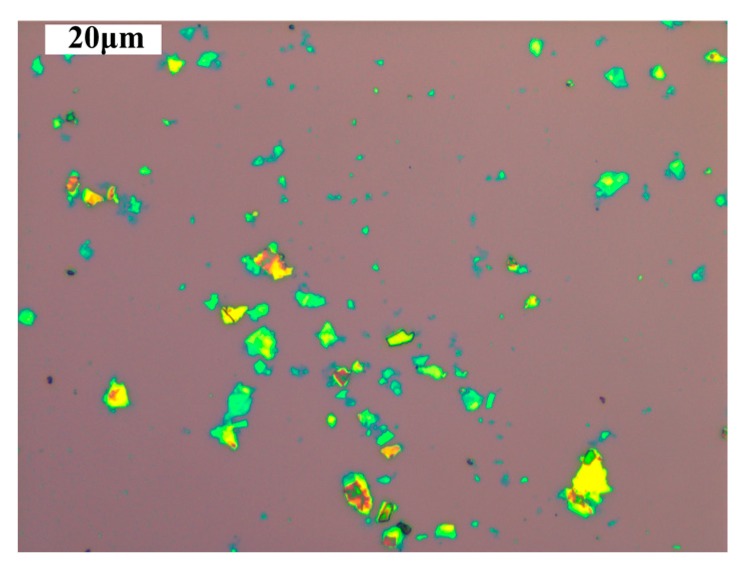
Optical image of the GO on the SiO_2_/Si.

Figure 1 shows the optical image of the graphene on the silicon wafer covered by a 200 nm-thick thermally grown silicon dioxide layer. Different layers of graphene exhibit the different optical contrast due to light interference effect of graphene on the silicon dioxide layer, so that we can distinguish between different layers and determine the boundaries of graphene. As shown the figure, the thickness of GO is extremely uniform, and the transverse size is in the micron order. 

**Figure 2:**
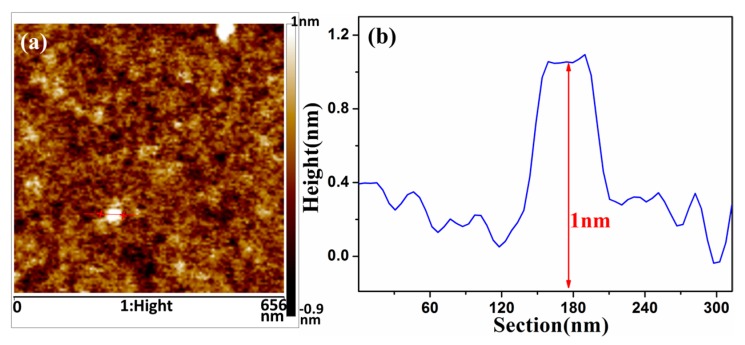
(**a**) Topographic atomic force microscope image of the GO; and (**b**) corresponding height profiles.

Figure 2a is an atomic force image of GO on the silicon wafer, and Figure 2b describes the height profile of the straight line in Figure 2a. As shown in the pictures, the thickness of GO is about 1 nm. Due to the function of the oxidation agent, hydroxyl, carboxyl, epoxy and carbonyl groups have appeared on the surface of graphene in the process of GO preparation. Because of the existence of these functional groups, GO exhibits a good hydrophilicity and can be stably dispersed in aqueous solution. In addition, the monolayer thickness of intrinsic graphene is 0.34 nm, but the thickness of GO increases because of the existence of surface functional groups.

**Figure 3:**
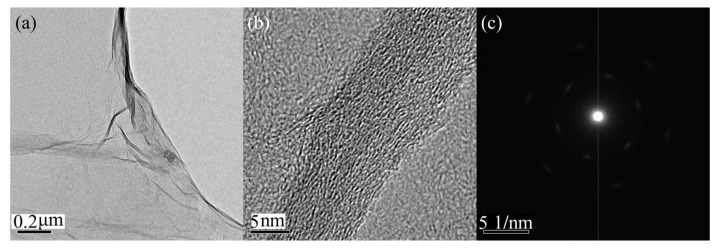
(**a**) Low magnification TEM image of the GO on the micro grid; (**b**) high resolution transmission electron microscopy image of the GO; and (**c**) the selected area electron diffraction pattern of the GO.

Figure 3 is the TEM image of GO. The dispersion of GO is added to the micro grid after being diluted, and then tested after drying. It can be found from the TEM image that graphene presents a translucent state, indicating that GO we prepared own ultrathin properties which is consistent with the results measured by AFM. Also we can see there are a lot of shrinkages and wrinkles on the surface of GO, which is the production of GO in order to keep its own thermodynamic stability. There is no such graphene with not rugged but strictly smooth surface. It is observed that it has a curl at the edge of the graphene and shapes into a thick edge with obvious optical contrast. The curl and fold of the graphene makes the originally tiled crystal surface upright so that the thickness of graphene can be easily observed by transmission electron microscopy. Figure 3b is a high resolution transmission electron microscopy image of the curl edge of graphene. It can be seen clearly that the thickness of GO is about 1 nm, which is consistent with the characterization of AFM. Figure 3c is the selected area electron diffraction image of graphene along the crystallographic axis of [001] which is perpendicular to the surface of graphene. As can be seen from the figure, graphene itself has a six-fold symmetrical structure, which is related to its hexagonal structure. The electron diffraction pattern consists of two sets of diffraction spots: the six diffraction spots of the inner surface correspond to the $\{10\bar{1}0\}$ crystal plane of graphene, and the other six diffraction spots of the outer surface correspond to the $\{11\bar{2}0\}$ crystal plane.

**Figure 4:**
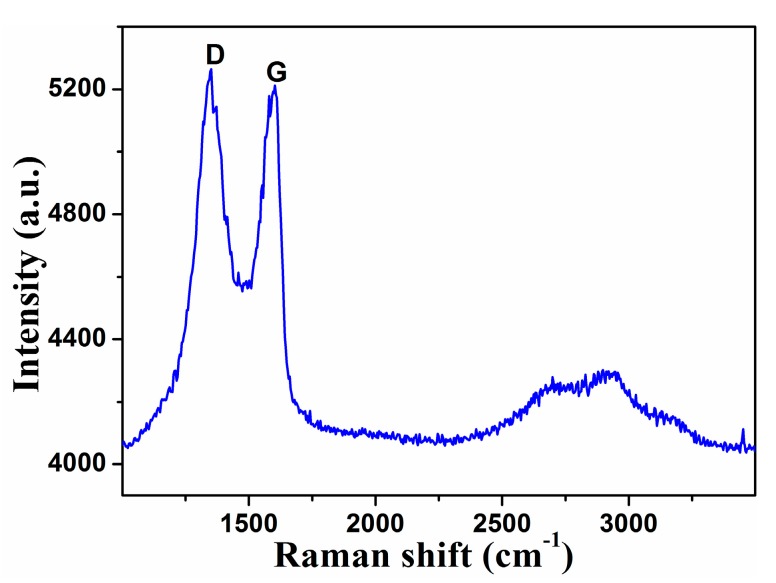
Raman spectrum of GO.

Raman spectroscopy has been widely used in the characterization of two-dimensional nanomaterials. For graphene, the Raman signal will change significantly with the alteration of the number of layers. Then, informations of graphene species, number of layers and others can be determined by observing the change of Raman signals. The dispersion of GO is added to the silicon wafer with 200 nm silicon oxide on the surface, and then the Raman spectrum is measured after vacuum drying. Confocal Raman spectrometer of Witec alpha 300R with optical fiber laser of 532 nm wavelength is used to test the Raman spectra of GO. In order to avoid the damage of the graphene, the laser energy is below 1 mW. It can be observed from Figure 4 that GO has two Raman peaks. One is the D peak at 1350 cm^−1^, which originates from the breathing vibration of *sp*^2^ atoms in the carbon ring and belongs to the A_1g_ symmetry mode. Usually the D peak is forbidden, and its intensity in graphite powders is very weak. However, due to the presence of surface disorder, especially the defects, the symmetry of the GO is broken, so that the vibration is permitted. Another peak is the G peak at 1580 cm^−1^. The G peak with the symmetry of E_2g_ originates from the first order Raman scattering process. In addition to the D peak and G peak, there is still a 2D peak at the frequency of 2680 cm^−1^. However, because of a large number of defects on the surface, the 2D peak cannot be observed in GO.

### 2.2. Experimental Setup

**Figure 5:**
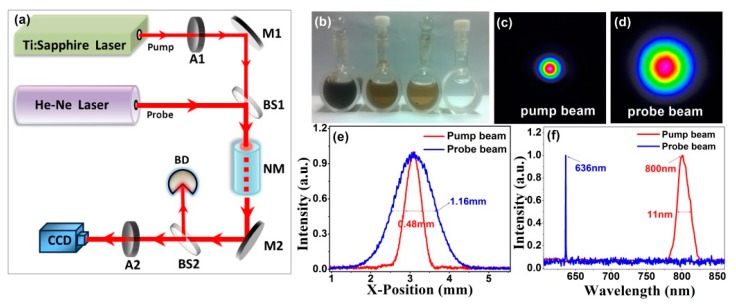
(**a**) Experimental scheme for spatial light modulation based on plasma channels. M1, silver-coated plane mirror; A1 and A2, attenuators; BS1 and BS2, beam splitters; NM, nonlinear material; (**b**) four kinds of nonlinear samples; (**c**–**f**) the intensity profiles and spectrums of pump and probe beams.

Figure 5a shows our experimental setup for spatial light modulation based on plasma channels. We use a Ti:sapphire amplified laser system as pump lasers source, it delivers 126 fs pump pulses with a central wavelength of 800 nm at a repetition rate of 1 kHz. In the experiment, pump pulses are used for the generation of plasma channels. The probe beam (He-Ne laser beam) is used as modulated beam. Figure 5b displays four types of samples in the experiment, from right to left in the order: distilled water, GO dispersion solutions in water with a concentration of 625 μg/mL, 1.25 mg/mL and 2.5 mg/mL. Figure 5c,d display initial spatial intensity profiles of two beams, and their corresponding cross lines (*y* = 0) are shown in Figure 5e. Profiles of pump and probe beams are nearly Gaussian, with full width at half maximum (FWHM) values of 0.48 and 1.16 mm, respectively. Figure 5f shows the spectrum of two beams, it is easy to see that the bandwidth of pump lasers is 12 nm. We use an attenuator (A1) for regulating the input power of the pump beam. Beam splitters BS1 is a dichroic mirror coated to have high reflectivity at 636 nm and high transmission at 800 nm. After passing through BS1, the pump beam and probe beam are spatially overlapped. And then, they propagate collinearly through a nonlinear material NM (GO dispersion solution in distilled water). After passing through BS2, the probe beam is separated form pump beam and is directed to a CCD camera, the pump beam is directed to a beam dump (BD) reflected by BS2. A high-resolution CCD camera is set to capture images of probe beams, so we can obtain the spatial modulation on the probe beam in real time. The adjustable attenuator A2 is customized to protect the CCD camera from damage caused by high-powered laser beams.

## 3. Results and Discussion

**Figure 6:**
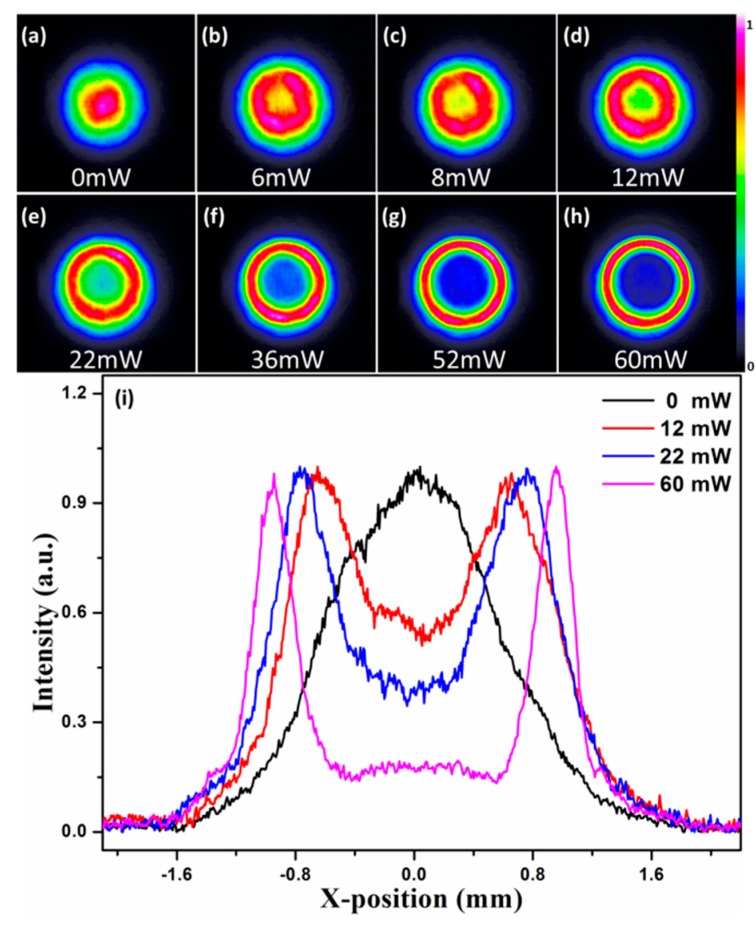
Spatial intensity distributions of probe beam at different *P*_fs_ and corresponding cross lines (*y* = 0) when *P*_fs_ is tuned.

Figure 6 displays the spatial modulation on the probe beam in the GO dispersion solution at concentration of 2.5 mg/mL. When the power of the pump beam (*P*_fs_) is 0 mW, the probe beam keeps its initial spatial intensity distribution. Increasing the power to 6 mW, it is easy to see from Figure 6b that a dark spot appears at the center of the probe beam. That is to say, the probe beam is modulated into a beam with a dark spot. This phenomenon can be attributed to the effect of a graded-index plasma lens. As femtosecond pulses propagate in a GO dispersion solution, the giant optical nonlinearity of GO could play an important role in the formation of plasma channels, plasma channels are generated when *P*_fs_ is above the ionization threshold. Plasma channels will persist during the experimental session because of femtosecond pulses sequences. Electron density and refractive index distributions in the generated plasma channel are close to Gaussian because of a Gaussian intensity profile of the pump beam. Lasers can only propagate in the plasma where the electron density is below the critical value. The probe beam cannot pass through the center region of plasma channels because of a high electron density of the plasma. According to the Fermat principle, the probe beam is focused when it passing through the periphery of plasma, while the plasma center is defocusing. Similar to a beam passing through a graded-index diverging lens, the probe beam is deflected when it propagates in the periphery of the plasma. The probe beam cannot pass through the center plasma; hence, a circular dark spot at the center region of the probe beam is formed. We only experimentally display the Gaussian distribution of the pump laser intensity, but our method has general character and other non-Gaussian conditions can also be analyzed by this method. If the spatial profile of pump beam can be adjusted conveniently, it is expected that the probe beam is modulated into a beam with any spatial distribution.

Increasing *P*_fs_ continuously (8 mW to 60 mW), the spatial intensity of probe beams at the central zone always decreases and the dark spot size increases. In other words, the modulated area of the probe beam enlarger gradually as *P*_fs_ increases. If we continue to increase *P*_fs_, it is expected that the spatial intensity at the center zone reduce to zero and probe beam is modulated into a ring-shaped beam. We can explain the reason as follows. The region in the plasma where probe beams can pass through ($n_{e}<n_{c}$) decreases as *P*_fs_ increases. The increase of *P*_fs_ results in a larger electron density gradient and refractive index gradient, leading to that the defocusing of the plasma center and the focusing of the plasma periphery become more evident. Hence, a more prominent spatial modulation on the probe beam is formed. If *P*_fs_ is set to zero, the spatial modulation on the probe beam disappears immediately and it recovers its initial beam profile in real time. So, we can easily control the spatial modulation on the probe beam in real time by adjusting *P*_fs_.

**Figure 7:**
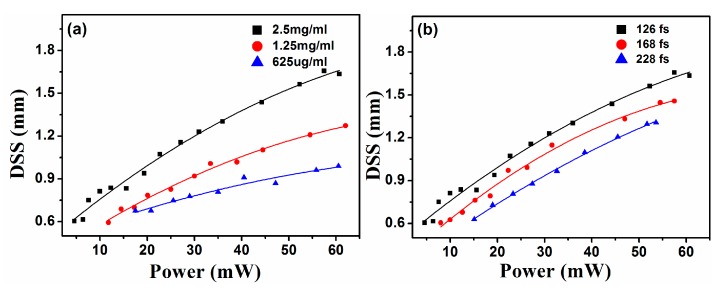
Relationships among DSS of modulated beam, concentration of GO dispersion solution, power and pulse-width of pump lasers.

Then we quantitatively analyze relationships among degree of modulation and dispersion solutions concentration, pulse width of pump lasers, and they are shown in Figure 7. Figure 7a shows relationships between *P*_fs_ and DSS of modulated beam generated in three GO dispersion solutions (2.5 mg/mL, 1.25 mg/mL, and 625 μg/mL). DSS is defined as the FWHM of the radial-intensity distribution inside the notch of the modulated beam. Simlar to Figure 6, Figure 7a displays visually that DSS of modualted beam increases as *P*_fs_ increase. Namely, spatial modulation on the probe beam is gradually enhanced as *P*_fs_ increase. To generate a same spatial modulation (a same DSS) on the probe beam, the power required in high concentration GO dispersion solutions is lower than that of in low concentration dispersion solutions. For example, an modulated beam with DSS = 0.9 mm will be produced in the dispersion solution at a concentration of 2.5 mg/mL when *P*_fs_ is 15 mW, while the power is 46 mW when the concentration is decreased to 625 μg/mL. From a simple physical standpoint, we understand the phenomenon as follows. When pump pulses propagate in a GO dispersion solution, nonlinear effect is enhanced as concentration increases, it is more easily and sensitive to generate a plasma channel. The power required for the generation of a same plasma channel in the low concentration solution is higher than that of in a high concentration solution. So, in order to achieve a same spatial modulation, the needed power is inversely proportional to the concentration of GO dispersion solutions. That is to say, it is easily to obtain a more prominent spatial modulation in the high concentration GO dispersion solution. Figure 7b displays relationships between *P*_fs_ and DSS of modulated beam under three pulse-widths of pump lasers. From Figure 7b, for achieving a same spatial modulation, the required power is proportional to pulse-width of pump lasers. This is because that, under the condition of a same average power of pump beams, it is more easily to generate plasma channels with a smaller pulse-width. From above, high power and small pulse-width of pump pulses, high concentration GO dispersion solution are all favorable terms for spatial modulation on the probe beam.

**Figure 8:**
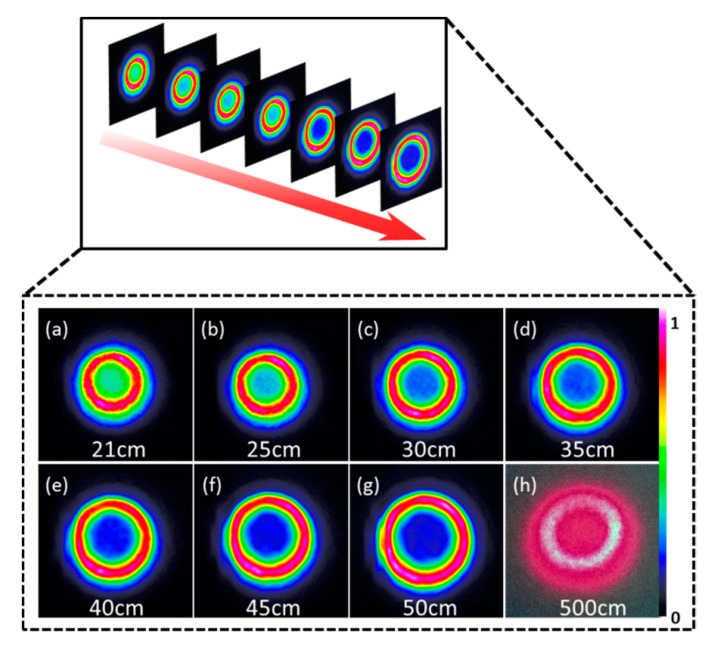
Propagations of modulated beams in the free space.

In the following, we study the propagation of the modulated beam in free space. CCD camera is set to move along the optical axis and then we capture a series of spatial intensity maps of modulated beams. Figure 8 shows intensity distributions of modulated beams generated in the GO dispersion solution (2.5 mg/mL) as a function of propagation distance D when *P*_fs_ is 30 mW. The propagation distance D is defined as the length between the glass cuvette and CCD camera. Because the size of modulated beam is beyond the sensing area of CCD when the distance is 500 cm, so we use a digital camera to capture the image of the modulated beam. Figure 8 vividly displays the propagation of the modulated beam. It is easy to see that the modulated beam keeps its initial beam spatial profile almost invariant and exhibits excellent propagation properties in free space. So, the way using GO for spatial light modulation is feasible and excellent. 

**Figure 9:**
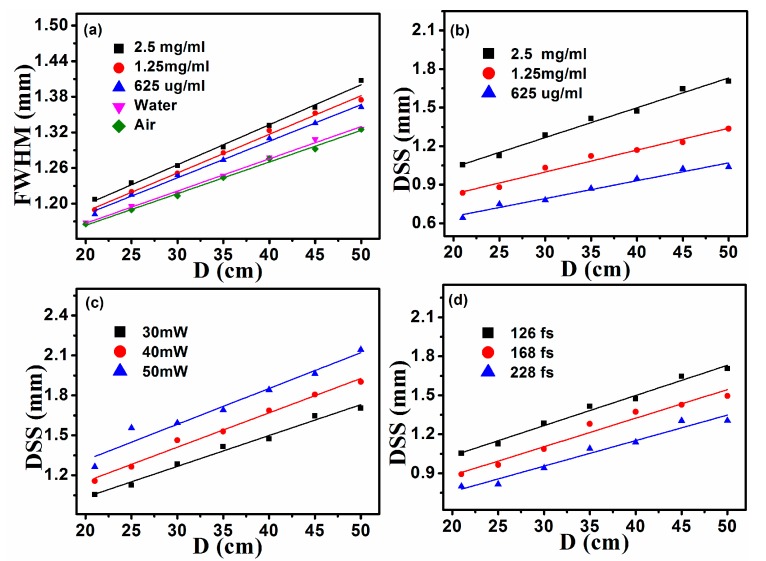
(**a**) Beam widths of probe beams after passed through different medium at different linear propagation distance D; (**b**–**d**) relationships between DSS of modulated beams and propagation distance D with different concentrations, *P*_fs_ and pulse-width of pump lasers.

To further quantitatively investigate the divergence of the modulated beam in the free space, initial probe beams are firstly set to pass through air, water and three types of GO dispersion solutions (2.5 mg/mL, 1.25 mg/mL and 625 μg/mL). Their beam widths at different propagation distance are displayed in Figure 9a. There are no plasma channels existed in the absence of ionization by pump beams. So the probe beam keeps its initial spatial distribution. Beam widths of initial probe beams after passed through GO dispersion solutions are larger than that of water and air. Moreover, beam widths of initial probe beams all increase with the increase of D. Due to the linear refractive index of dispersion solutions increases with the increase of the concentration, we can see that degree of divergence of the beam width increases as the concentration increase.

Figure 9b–d show the divergence of modulated beams generated in GO dispersion solutions at different *P*_fs_, pulse widths of the pump lasers, concentrations of dispersion solutions. Specifically, Figure 9b displays relationships between propagation distance D and DSS of modulated beams at three concentrations when *P*_fs_ is 30 mW (pulse-wdth ~ 126 fs). Results depicted in Figure 9b agree with conclusions of Figure 7a, it is more easily to achieve the spatial modulation on the probe beam at high concentrations of GO dispersion solutions. For a GO dispersion solution (2.5 mg/mL), DSS of modulated beams increases by 61.6% when D is increased from 20 cm to 50 cm, it is 61.9% for 625 μg/mL GO dispersion solution. Almost no difference existed among the divergence of modulated beams generated in three kinds GO dispersion solutions. Based on the same analysis, we can get informations directly from Figure 9c,d, the divergence of modulated beams at different *P*_fs_, pulse widths of pump lasers are nearly the same. They are the same as depicted in Figure 6 and Figure 7, it is more easily to obtain the spatial modulation on the probe beam at high power or small pulse-width of pump lasers. Through the above description, it finds that the divergence of modulated beams based on GO is not relevant to power and pulse-width of pump lasers, dispersion solution concentration. 

## 4. Conclusions

In conclusion, we successfully synthesize and characterize GO, due to the large amount of functional groups on the surface, the GO is hydrophilic so that it can be dispersed in water, and the thickness of fewer layer oxide graphene has been increased to 1 nm. Then, we experimentally demonstrate the spatial light modulation on the probe beam based on plasma channels generated in the GO dispersion solution. In additional, the propagation of modulated beams in the free space is studied. It finds that the modulated beam keeps its initial beam spatial profile almost invariant and exhibits excellent propagation properties. The divergence of modulated beams is not relevant to power and pulse-width of pump lasers, the concentration of GO dispersion solutions. We can easily control the spatial modulation on the probe beam by adjusting power and pulse-width of pump lasers, dispersion solution concentration.
